# p53 Interacts with VDAC1, Modulating Its Expression Level and Oligomeric State to Activate Apoptosis

**DOI:** 10.3390/biom16010141

**Published:** 2026-01-13

**Authors:** Elinor Gigi, Aditya Karunanithi Nivedita, Danya Ben-Hail, Manikandan Santhanam, Anna Shteinfer-Kuzmine, Varda Shoshan-Barmatz

**Affiliations:** 1 Department of Life Sciences, Ben-Gurion University of the Negev, Beer Sheva 84105, Israelnivedita@post.bgu.ac.il (A.K.N.); santhana@post.bgu.ac.il (M.S.); 2 National Institute for Biotechnology in the Negev, Ben-Gurion University of the Negev, Beer Sheva 84105, Israel; shteinfe@post.bgu.ac.il

**Keywords:** apoptosis, mitochondria, oligomerization, p53, VDAC1

## Abstract

The p53 tumor suppressor, a key transcription factor, acts as a cellular stress sensor that regulates hundreds of genes involved in responses to DNA damage, oxidative stress, and ischemia. Through these actions, p53 can arrest cell cycle, initiate DNA repair, or trigger cell death. In addition to its nuclear functions, p53 can translocate to mitochondria to promote apoptosis. Studies using isolated mitochondria have suggested that p53 drives the voltage-dependent anion channel (VDAC1) into high molecular mass complexes to mediate apoptosis. VDAC1 is a central regulator of cellular energy production and metabolism and also an essential player in apoptosis, induced by various apoptotic stimuli and stress conditions. We previously demonstrated that VDAC1 oligomerization, induced by various apoptosis stimuli and stress conditions, forms a large pore that enables cytochrome *c* release from mitochondria, thereby promoting apoptotic cell death. In this study, we show that p53 interacts with VDAC1, modulates its expression levels, and promotes VDAC1 oligomerization-dependent apoptosis. Using purified proteins, we found that p53 directly binds VDAC1, as revealed by microscale thermophoresis and by experiments using bilayer-reconstituted VDAC1, in which p53 reduced VDAC1 channel conductance. Furthermore, overexpression of p53 in p53-null cells or in cells expressing wild-type p53 increased VDAC1 expression and induced VDAC1 oligomerization even in the absence of apoptotic stimuli. Together, these findings identify VDAC1 as a direct p53 target whose expression, oligomerization, and pro-apoptotic activity are regulated by p53. They also reinforce the central role of VDAC1 oligomerization in apoptosis.

## 1. Introduction

The tumor suppressor protein p53 is a transcription factor that is induced by stress signals, such as DNA damage, oncogene activation, and nutrient deprivation [1]. It plays a central role in the regulation of the cell cycle, apoptosis, and senescence, acting through various transcription-dependent and -independent mechanisms [2,3,4]. Thus, apoptosis and cell-cycle arrest are the two main outcomes of p53 activation [5,6].

Due to its central role in activating cellular responses to stress, p53 is frequently absent or mutated in cancerous cells, leading to cell phenotypes that are resistant to chemotherapy [7]. The outcome of the p53-mediated stress response depends on the cell type, as well as the extent, duration, origin, and type of cellular stress, with activation of p53 leading to cell-cycle arrest and DNA repair [4]. Following its induction, p53 binds specific promoters in the DNA and activates the transcription of a wide array of target genes, aimed at eliminating the danger of potential cancer [8,9].

p53-controlled apoptosis involves the transcriptional induction of components of the death receptor and mitochondrial pathways, including CD-95, PUMA, Noxa, Bax, and others, which cooperatively promote cell death [10,11]. At the same time, p53 has also been reported to possess cytosolic functions, such as inhibiting autophagy [12,13,14], or interacting with Bcl-2 family members to promote apoptosis [15]. Protein binding partners of p53 play a crucial role, as they not only contribute to p53 activation in response to cellular stress, but are also involved in p53 stabilization [16].

Wild-type p53 has also been shown to accumulate in the cytoplasm of some tumors [17,18]. The translocation of cytoplasmic p53 to mitochondria in response to a stress signal has been demonstrated in several studies [19,20,21,22,23,24]. p53 translocated to the mitochondria induces cytochrome *c* (Cyto *c*) release and apoptosis, as well as the activation of caspases via interactions with the anti- and pro-apoptotic Bcl-2 family of proteins to either inhibit or activate these proteins [19,20,21,22,23]. In addition, p53 can directly promote mitochondrial outer membrane permeabilization (MOMP), leading to apoptosis by modulating the actions of Bcl-2 family members [15,23]. Studies with recombinant p53 and isolated unstressed mitochondria in cell-free systems revealed that wild-type p53, but not tumor-derived missense mutants induced the oligomerization of Bak and Bax, and MOMP activity [25,26,27,28]. Yet, using mitochondria isolated from *PUMA*^−/−^ or *Bax*^−/−^ mice, p53 mitochondrial translocation and release of Cyto *c*, SMAC/Diablo, and AIF were obtained independent of PUMA and Bax [27]. A role for p53 in activating necrosis has also been proposed, where in response to oxidative stress, the protein accumulates in the mitochondrial matrix and triggers mitochondrial permeability transition pore (PTP) opening and necrosis by physical interaction with the PTP regulator cyclophilin D [29]. Furthermore, it was proposed that the voltage-dependent-anion channel (VDAC) protein is a putative partner of p53 [29,30].

VDAC1, found in the outer mitochondrial membrane (OMM), assumes a crucial position in the cell, serving as the main interface between mitochondrial and cellular metabolisms, providing the passage for anions, Ca^2+^ and other cations, adenine nucleotides, and other metabolites into and out of the mitochondria, thus, playing a major role in regulating the metabolic and energetic functions of mitochondria [31,32,33]. Importantly, VDAC1 functions as an anchor point for mitochondria-interacting proteins involved in both cell survival and cell death pathways [31,34,35]. These include cytosolic, ER, and mitochondrial proteins that collectively regulate metabolic and signaling pathways and coordinate mitochondrial functions with other cellular activities [34,35].

Substantial evidence points to VDAC1 as being a key player in apoptosis, regulating the release of apoptogenic proteins from the mitochondria, such as Cyto *c*, and interacting with anti-apoptotic proteins [31,34,35] and other proteins regulating cellular processes [36].

Recently, we demonstrated that VDAC1 oligomerization is a general mechanism common to numerous apoptogens that act via different initiating cascades, and we proposed that a VDAC1 oligomeric structure forms a protein-conducting channel large enough for passage of a folded protein, such as Cyto *c* [37,38,39,40,41,42]. We demonstrated that VDAC1 oligomerization is coupled to Cyto *c* release and apoptotic cell death, as induced by various stimuli [37,38,39,40,41,42]. Moreover, it was shown that various apoptotic stimuli [43], including cisplatin [44], arbutin [45], UV irradiation and reactive oxygen species [46], etoposide [47], erastin [43,48,49,50,51,52,53,54,55,56,57,58,59,60,61,62,63,64], and more, upregulate VDAC1 expression levels, resulting in VDAC1 oligomerization and apoptosis. Thus, we further suggest that the activities of numerous anti-cancer drugs and treatments are mediated via regulating VDAC1 expression levels, and that VDAC1 expression levels are associated with many diseases [48,49,50,51,52,53,54,55,56,57,58,59,60,61,62,63,64]. Furthermore, we recently demonstrated a tight correlation between increases in VDAC1 expression levels, VDAC1 oligomerization, and apoptosis [41,43,48,49,50,51,52,53,54,55,56,57,58,59,60,61,62,63,64]. Accordingly, we suggest that apoptotic stimuli act by enhancing VDAC1 expression levels, and lead to the formation of VDAC1 oligomers, allowing the release of mitochondrial pro-apoptotic proteins, thereby activating apoptosis.

Protein binding partners of p53 play a crucial role as they not only contribute to p53 activation in response to cellular stress, but are also involved in p53 stabilization [16]. p53 has been shown to regulate VDAC1 expression in neuronal cells through mechanisms involving reduced ATP levels and metabolic oxidative stress [65]. In addition, it has been previously proposed that p53 promotes VDAC1 oligomerization into high molecular weight complexes [27,29]. Here, we demonstrate that p53 interacts with VDAC1 and, when expressed in cells, promotes VDAC1 oligomerization. Additionally, p53 overexpression resulted in upregulation of VDAC1 expression levels and enhanced VDAC1 oligomerization and apoptosis in the absence of apoptotic stimuli. Moreover, p53-induced apoptosis was prevented in cells silenced for VDAC1 expression or in the presence of the VDAC1-interacting molecule DIDS. These findings point to VDAC1 as a target of p53 and further support VDAC1 oligomerization as a key step in the induction of apoptosis.

## 2. Materials and Methods

### 2.1. Materials

4,4′-diisothiocyanostilbene-2,2′-disulfonic acid (DIDS), Diamidino-2-phenylindole (DAPI), leupeptine, phenylmethylsulfonyl fluoride (PMSF), propidium iodide (PI), bovine serum albumin (BSA), trypan blue, triton X-100, and dimethyl sulfoxide (DMSO) were purchased from Sigma-Aldrich (St. Louis, MO, USA). si-RNA against VDAC1 was from Gene Pharma (Suzhou, China). Jet PRIME was from PolyPlus Transfection (Illkirch, France). Ethylene glycol-bis (succinimidylsuccinate) (EGS) was obtained from Pierce Chemical (Rockford, IL, USA). Paraformaldehyde was purchased from Emsdiasum (Hatfield, PA, USA). MitoTracker Red was acquired from Thermo Fisher Scientific (Waltham, MA, USA). Annexin V-fluorescein isothiocyanate (FITC) was from Enzo Life Sciences (Lausen, Switzerland). si-RNA against p53 was obtained from Santa Cruz Biotechnology (Santa Cruz, CA, USA). Dulbecco’s modified Eagle’s medium (DMEM) and Roswell Park Memorial Institute medium (RPMI 1640) were purchased from Gibco (Grand Island, NY, USA). Fetal calf serum, L-glutamine, and penicillin-streptomycin solution were purchased from Biological Industries (Beit Haemek, Israel). Fluoroshield was obtained from Immuno Bio Science Corporation (Washington, DC, USA).

Primary and secondary antibodies, their source, and the dilutions used are detailed in Table 1.

### 2.2. p53 Expression and Purification

A plasmid encoding for full-length super-stable p53 (FL-p53, residues 1–393) was kindly obtained from Dr. Azem Abdussalam, Tel Aviv University (Tel Aviv, Israel) and was cloned into the pET24a-HLTV vector to yield a protein containing an N-terminal 6xHis/lipoamyl domain/TEV protease cleavage site domain. FL-p53 is a quadruple mutant form (M133L/V203A/N239Y/N268D) of the protein, which was stabilized by the mutations, as described previously [66,67,68]. The vector was transformed into *Escherichia coli* Rosetta cells for overexpression. To produce FL-p53, the transformed cells were grown in 650 mL of 2xYT medium containing 50 μg/mL kanamycin and 34 μg/mL chloramphenicol at 37 °C. When A600 reached 0.6, zinc sulfate was added for a final concentration of 0.1 mM, and protein expression was induced with 0.4 mM isopropyl β-D-1-thiogalactoside. The cells were grown at 23 °C for an additional 3 h and then harvested by centrifugation (15 min at 3000× *g*, 4 °C). The cells were lysed by sonication in 50 mM KPi buffer (pH 8; 300 mM NaCl, 15 mM β-mercaptoethanol, 1 μg/mL leupeptin, and 150 μM PMSF). The cell extract was centrifuged, and soluble p53 was purified by chromatography using nickel-nitrilotriacetic acid resin on a 1 mL column (HisTrap HP, GC). p53 was eluted from the column by a linear gradient of imidazole (10–250 mM) in 50 mM KPi buffer, pH 8. The eluted protein was dialyzed against buffer containing 25 mM NaPi pH 7.5; 300 mM NaCl, 5 mM DTT, and 10% glycerol. The dialyzed sample was incubated with the TEV protease (1:15 mg/mg, 16 h) to remove the N-terminal 6xHis/lipoamyl domain. After dialysis, a second Ni-affinity chromatographic step was carried out to separate the 6xHis/lipoamyl domain from the FL-p53. Purified p53 in 50 mM Pi, pH 8, 300 mM NaCl, 15 mM β-mercaptoethanol, 5 mM DTT, and 10% glycerol was stored in aliquots at −80 °C. Protein concentration was determined using the Lowry method.

### 2.3. Exchange Buffer Using Centrifugation-Chromatography Method

p53 was separated from the reducing agents DTT and β-mercaptoethanol reagents by centrifugation-chromatography on a column of Sephadex G-50 fine filtration resin, equilibrated with PBS, pH 7.4.

### 2.4. Cell Lines and Transfection

HeLa (human cervical adenocarcinoma), A549 (non-small human lung carcinoma), and H358 (human non-small cell lung cancer) cell lines were obtained from ATCC and maintained in DMEM or RPMI, respectively. Mediums were supplemented with 10% fetal calf serum, 2 mM l-glutamine, 100 μg/mL penicillin, 100 μg/mL streptomycin, and non-essential amino acids. All cells were maintained in a humidified atmosphere at 37 °C with 5% CO_2_. Logarithmically growing cells were transfected with empty or pCMV-, p53- or p53-GFP-encoding plasmids (Addgene #12091; MIT, Boston, MA, USA) using Jet-Prime transfection reagent.

### 2.5. Apoptosis Analysis

Following treatment with the indicated reagents, cells were analyzed for apoptotic cell death upon staining with PI and annexin V-FITC, which was carried out according to the manufacturer’s instructions with minor modifications. After treatment, cells were harvested (1500× *g*, 5 min), washed, and re-suspended in 200 µL of binding buffer (10 mM Hepes/NaOH, pH 7.4, 140 mM NaCl, and 2.5 mM CaCl_2_). Annexin V-FITC was added, and the cells were incubated for 30 min, protected from light. The cells were then washed once with the binding buffer and re-suspended in it, to which PI (final concentration of 3.75 μg/mL) was added immediately before flow cytometry measurements. At least 10,000 events were collected, recorded on a dot plot, and analyzed by flow cytometry with an iCyt sy3200 Benchtop Cell Sorter/Analyzer (Sony Biotechnology Inc., San Jose, CA, USA) and analysis with EC800 software (version 1.3.7).

### 2.6. Chemical Crosslinking

Cells were cultured in 6-well plates to ~60% confluence, subjected to the desired treatments, harvested, and washed with PBS. Protein concentrations were determined using the Lowry method. To assess VDAC1 oligomerization, cells (1.5–3 mg/mL protein in PBS, pH 8.3) were incubated with or without the crosslinking agent EGS (20–300 μM) for 15 min at 30 °C. Cell lysates were then prepared, and protein concentrations were determined. Samples (60–90 μg) were analyzed by SDS–PAGE and transferred to nitrocellulose membranes for immunoblotting with anti-VDAC1 antibodies. Before immunoblotting, membranes were treated with 0.1 M glycine (pH 2.2) and subsequently washed several times with Tris-buffered saline containing 0.1% Tween-20 (TBST). Quantification of immunoreactive VDAC1 dimers, trimers, and multimers was performed using ImageJ software (version 1.54p).

### 2.7. VDAC1 Silencing and Transfection

VDAC1 specific human si-RNA (h)VDAC1, and non-targeting si-RNA (si-NT) were synthesized and obtained from Gene Pharma (Suzhou, China).

si-NT, Sense: 5′ GCAAACAUCCCAGAGGUAU3′

Anti-sense: 5′ AUACCUCUGGGAUGUUUGC3′

si-(h)VDAC1, Sense: 5′ ACACUAGGCACCGAGAUUA 3′

Anti-sense: 5′ UAAUCUCGGUGCCUAGUGU 3′

Cells were seeded (40–60% confluence) in 6-well plates and transfected with 50 or 100 nmol/L si-(h)VDAC1 using JetPRIME transfection reagent, according to the manufacturer’s instructions. Post transfection, cells were subjected to immunoblotting for VDAC1 expression levels using anti-VDAC1 specific antibodies.

### 2.8. Immunofluorescence

A549 cells were seeded on 13 mm glass coverslips and transfected with an empty or p53-encoding plasmid (1–2 μg). Then cells were washed with PBS and fixed with 4% paraformaldehyde at room temperature for 15 min, incubated for 1 h with blocking solution [5% normal goat serum (NGS) and 0.1% Triton X-100 in PBS], and incubated with primary antibodies (sources and dilutions used are detailed in Table 1) for 1.5 h. After washing with PBS, the cells were incubated for 1.5 h with fluorophore-conjugated anti-mouse or anti-rabbit secondary antibodies (Table 1) in the dark. Then nuclei were stained with DAPI (0.07 μg/mL) for 15 min, and carefully washed, dried, and mounted on slides with Fluoroshield mounting medium (Immunobioscience, Mukilteo, WA, USA). After overnight drying at 4 °C, images were acquired using a confocal microscope (Olympus 1X81, Tokyo, Japan).

### 2.9. Cell Lysate Preparation

Cells were harvested, centrifuged for 5 min at 1500× *g*, washed twice with PBS and re-suspended with lysis buffer (100 mM tris/HCl (pH 8.0), 5% SDS), supplemented with a protease inhibitor cocktail (Calbiochem, San Diego, CA, USA). Samples were then sonicated for 10 s, centrifuged (15,000× *g*, 10 min at 4 °C), the protein concentration of supernatant was determined according to a Lowry assay.

### 2.10. Gel Electrophoresis and Immunoblot Analysis

For electrophoresis, samples were diluted 1:4 with a 4× sample buffer containing 40% glycerol, 4% β-mercaptoethanol, 8% SDS, 0.26 M tris, pH 6.8, and bromophenol blue, and incubated for 10 min at 70 °C. SDS-PAGE was performed according to the Laemmli protocol [69]. Protein aliquots (15–30 μg) were subjected to SDS-PAGE and then were electro-transferred onto nitrocellulose membranes for immunostaining. The membranes were incubated with a blocking solution containing 5% non-fat dry milk and 0.1% Tween-20 in Tris-buffered saline (TBST), followed by incubation with primary antibodies (Table 1). Subsequently, membranes were incubated with HRP-conjugated anti-mouse or anti-rabbit IgG as secondary antibodies. Enhanced chemiluminescent substrate EZ-ECL (Biological Industries, Beit Haemek; Israel) was used to detect HRP activity. Band intensities were analyzed by densitometry using Image Gauge software (version 3.0; Science Lab 2001) provided by Fujifilm (Tokyo, Japan) or ImageJ (version 1.54p; Sun Microsystems, Redwood City, CA, USA) software, and the values were normalized to the intensities of the appropriate β-actin signal that served as a loading control. The indicated molecular mass of each protein was determined using the standard markers shown for the corresponding blot in the original images provided in the Supplementary Materials.

### 2.11. VDAC1 Purification and Reconstitution into a Planar Lipid Bilayer and Analysis of Channel Activity

VDAC1 purified from rat liver mitochondria as previously described [70]. Briefly, rat liver mitochondria (5 mg/mL) were suspended in 10 mM Tris-HCl (pH 7.2) and incubated with 2% LDAO at 0 °C for 20 min. The mixture was centrifuged at 14,000× *g* for 30 min, and the resulting supernatant was applied to a dry celite–hydroxyapatite (2:1) column. VDAC1 was eluted using a buffer containing 2% LDAO, 10 mM Tris-HCl (pH 7.2), 50 mM NaCl, and 22 mM NaH_2_PO_4_. The VDAC1-containing fractions were dialyzed against 10 mM Tris-HCl (pH 7.2) and further purified by chromatography on a carboxymethyl cellulose (CMC) column. VDAC1 was eluted with a solution containing 10 mM Tris-HCl (pH 7.2), 0.1% LDAO, and 500 mM NaCl. The purified VDAC1 fractions were collected and used for channel conductance and microscale thermophoresis (MST) assays.

For channel reconstitution, a planar lipid bilayer (PLB) was prepared from soybean asolectin dissolved in n-decane (30 mg/mL). Purified VDAC1 (1–2 ng) was added to the *cis* chamber containing 0.5 M NaCl and 10 mM Hepes, pH, 7.4. After one or more channels were inserted into the PLB, excess protein was removed by perfusing the *cis* chamber to prevent further channel incorporation. Currents were recorded by voltage-clamping using a Bilayer Clamp BC-525B amplifier (Warner Instruments, Hamden, CT, USA). Current was measured with respect to the *trans* side of the membrane (ground). The currents were low pass-filtered at 1 kHz and digitized on-line using a Digidata 1440-interface board and Clampex software (version 10.2; Axon Instruments, Union City, CA, USA).

### 2.12. Microscale Thermophoresis (MST) Measurements

MST analysis was performed using a Nano Temper Monolith NT.115 apparatus (Nano Temper Technologies, Munich, Germany), as described previously [71]. Purified VDAC1 and p53 were fluorescently labeled using a Nano Temper protein-labeling Kit BLUE (L001, Nano Temper Technologies, Munich, Germany). A constant concentration of fluorescently labeled VDAC1 (100 nM) was incubated with different concentrations of p53 (1.14 nM–18.75 μM) in PBS buffer or fluorescently labeled p53 (2 μM) was incubated with different concentrations of VDAC1 (33.3 nM–5.33 μM) in PBS buffer. After 15 min of incubation in the dark, 3–5 μL of the samples were loaded into MST-grade glass capillaries (Nano Temper Technologies), and thermophoresis was measured with a Nano Temper Monolith-NT115 system (20% light-emitting diode, 40–60% IR laser power).

### 2.13. Proximity Ligation Assay (PLA)

Proximity ligation assays (PLA) were performed using the Duolink^®^ In Situ Red Starter Kit (Sigma-Aldrich; St. Louis, MO, USA), following the manufacturer’s protocol, to assess protein–protein interactions between VDAC1 and p53 as described previously described [72]. Briefly, A549 cells were seeded on 35 mm coverslips and cultured for 24 h to approximately 60% confluency, cells were washed with PBS, fixed in 10% formaldehyde (in PBS) for 15 min at room temperature, and permeabilized with 0.1% Triton X-100 in PBS for 15 min. Following a PBS wash, cells were blocked for 1 h at 37 °C using Duolink^®^ Blocking Solution.

For detection of protein proximity, cells were incubated for 1 h at RT with primary antibody pairs: mouse anti-p53 (1:500) and rabbit anti-VDAC1 (1:1000). Cells were then treated with species-specific PLA probes (PLUS and MINUS), each conjugated to complementary oligonucleotides. Ligation and amplification were performed according to the kit instructions, and interaction signals were visualized as distinct red fluorescent dots using Texas Red-labeled detection reagents. Nuclei were counterstained with DAPI, and coverslips were mounted using Fluoroshield mounting medium (ImmunoBioScience, Mukilteo, WA, USA). Confocal images were acquired using an Olympus IX81 microscope (Olympus corporation; Tokyo, Japan). Quantitative analysis of PLA signals (puncta per cell) was conducted using ImageJ software (version 1.54p; Sun Microsystems, Redwood City, CA, USA), based on fluorescence intensity across the entire cell area.

### 2.14. Nuclear Fractionation

The procedure for separation into nuclear and cytoplasmic fractions was performed according to the manufacturer’s instructions, using a nuclear/cytosol fractionation kit (Biovision, Milpitas, CA, USA). Cytosolic and nuclear fractions were prepared from si-NT and si-(h)VDAC1 (75 nM) treated cells subjected to centrifugation (16,000× *g*, 10 min), and the supernatants (cytosolic fraction) and pellets (nuclear fraction) that were resuspended in the original volume were obtained. Samples were subjected to immunoblotting for p53 and VDAC1 and KI-67, using specific antibodies.

### 2.15. Statistics

Results are presented as the means ± SEM of at least three independent experiments. Significance of differences was calculated by the two-tailed Student’s *t*-test using the *t*-test function provided by Microsoft Excel (version 2510). Statistically significance is reported at *p* < 0.05 (*), *p* < 0.01 (**) or *p* < 0.001 (***).

## 3. Results

In this study, we investigated the interaction of p53 with VDAC1 and assessed the ability of p53 to modulate VDAC1 expression, oligomerization, and apoptosis.

### 3.1. p53 Directly Interacts with VDAC1 as Revealed by MST and Bilayer Reconstituted VDAC1 Channel Activity

**Figure 1:**
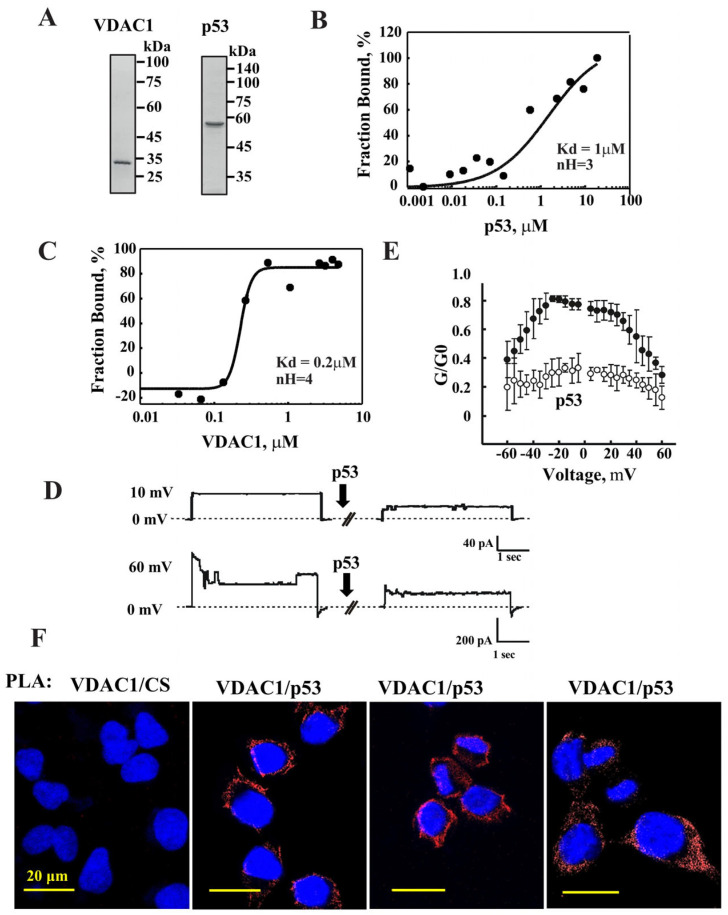
Purified VDAC1 directly interacts with purified p53 and reduces channel conductance of bilayer-reconstituted VDAC1. (**A**) Coomassie blue-stained SDS-PAGE profile of purified VDAC1 and p53 proteins. (**B**,**C**) MST measurements: purified VDAC1 (100 nM) (**B**) or p53 (2 μM) (**C**) were fluorescently labeled using a Nano Temper Protein-Labeling Kit BLUE and incubated 15 min with the indicated concentrations of p53 (1.14 nM–18.75 μM) or of VDAC1 (33.3 nM–5.33 μM), respectively. Then 3–5 μL of the samples were loaded into MST-grade glass capillaries, and thermophoresis was measured using a Monolith-NT115 apparatus. Data in B and C were analyzed using GraphPad Prism (version 10.6.1) to drive the binding constants (Kd) and Hill coefficients (nH). (**D**) Purified VDAC1 was reconstituted into a PLB and channel currents through it in response to voltage step 0–10 mV or 0–60 mV, recorded before and 5 min after the addition of p53 (0.5 μM). The dashed lines indicate zero current. (**E**) The effect of p53 on VDAC1 conductance as a function of voltage, from 60 mV to −60 mV. The average steady-state conductance at a given voltage (G) was normalized to the conductance at 10 mV (G0). The recordings were taken before (•) and 5 min after the addition of p53 (○) (n = 3). (**F**) Cells were subjected to in situ PLA to test for close association between VDAC1 (OMM) and p53 using specific antibodies. The ligation products appear in red; nuclei were DAPI-stained (blue). PLA using VDAC1 and matrix located citrate synthase (CS) are shown as negative control.

Previous studies have indirectly demonstrated the interaction between VDAC1 and p53 using purified p53 and isolated mitochondria [27,29,30]. Here, we analyzed the direct interaction between purified VDAC1 and p53 proteins (Figure 1A) using MST. MST offers a quantitative approach to assess protein interactions and allows for the determination of a binding affinity coefficient (Kd) [71]. Fluorescently labeled VDAC1 was incubated with increasing concentrations of purified p53 (1.14–18.75 μM) and MST analysis was performed (Figure 1B). The percentage change in normalized fluorescence (ΔF Norm, %) was plotted as a function of p53 concentration, yielding a dissociation constant (Kd) of 1 µM and a Hill coefficient (nH) of 3 (Figure 1B). These results suggest that VDAC1, likely in an oligomeric state, interacts cooperatively with at least three p53 molecules.

In a reciprocal MST experiment, fluorescently labeled p53 was incubated with increasing concentrations of purified VDAC1 (33.3 nM–5.33 μM). Analysis of the binding curve produced a Kd of 0.2 µM and a nH of 4 (Figure 1C). This experiment further indicates that VDAC1 interacts with three or more p53 molecules and vice versa. These findings are consistent with previous reports showing that both VDAC1 [37,38,39,40,41,42] and p53 [73] possess oligomeric structures.

Next, we examined the effect of p53 on the channel conductance of bilayer-reconstituted VDAC1 under voltage-clamp conditions (Figure 1D,E). The current produced in response to voltage steps from a holding potential of 0 mV–10 mV or 60 mV was recorded, and five min after the addition of purified p53, it was recorded again.

The recordings revealed that p53 significantly reduced VDAC1 channel conductance, stabilizing the channel in a low-conducting state (Figure 1D). VDAC1 is known to exhibit voltage-dependent gating, with maximal conductance typically observed between −20 mV and +20 mV, and reduced conductance at higher positive or negative voltages. In the presence of p53, the reduction in channel conductance was observed consistently across all voltage steps, indicating that p53 stabilizes VDAC1 in a low-conductance conformation regardless of the applied voltage gradient (Figure 1E).

These results further support a direct, functional interaction between p53 and VDAC1, with p53 modulating VDAC1 gating properties.

Next, to validate the direct interaction between p53 and VDAC1 in live cells, we performed an in situ proximity ligation assay (PLA), which enables visualization of protein–protein interactions at single-molecule resolution [74] (Figure 1F). Using anti-VDAC1 and anti-p53 antibodies, we detected a strong PLA signal, indicating close proximity between the two proteins at the mitochondria and confirming their interaction. This interaction is specific as no PLA signal was obtained between VDAC1 and citrate synthase, a mitochondrial matrix protein.

### 3.2. Subcellular Localization of p53 in Cells Silenced for VDAC1 Expression by Specific si-RNA

**Figure 2:**
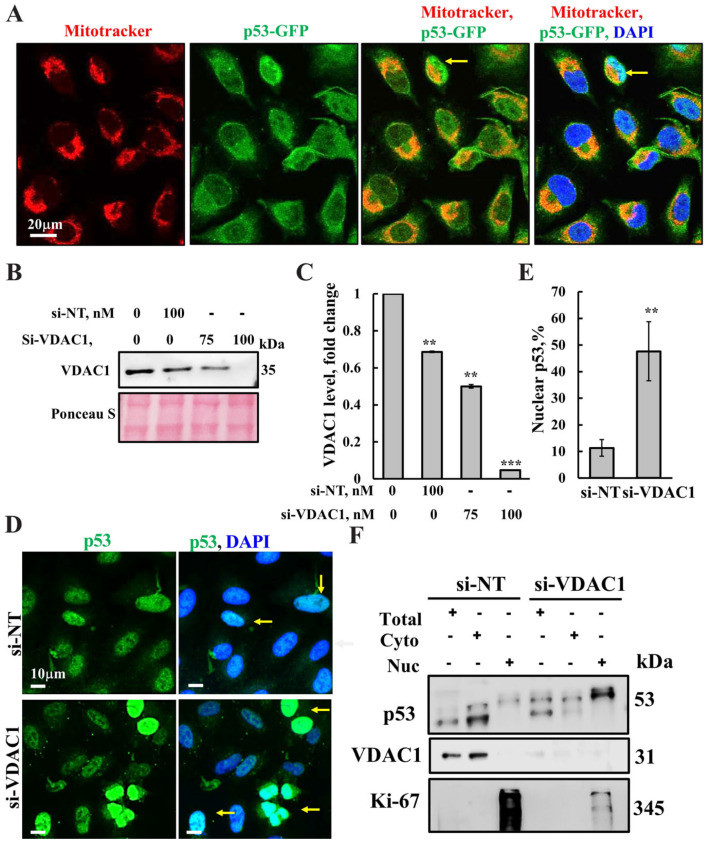
Silencing VDAC1 expression leads to p53 translocation to the nucleus. (**A**) HeLa cells were transfected to express p53-GFP 24 h post transfection. Cells were seeded on 35 mm coverslips cultured to a 60% confluency, stained with Mitotracker red (Invitrogen, Waltham, MS, USA), and imaged using a confocal microscope. Nuclei were stained with DAPI. Arrows point to the nuclear localization. (**B**) Control and si-NT- and si-(h)VDAC1-treated cells (100 nM si-NT or 75 or 100 nM of si-(h)VDAC1), 24 h post transfection cells were lysed and analyzed for VDAC1 expression level using immunoblotting and anti-VDAC1 antibodies. (**C**) Quantitative analysis of VDAC1 levels of the samples in (**B**). (**D**,**E**) p53 sub-cellular localization was analyzed in control cells transfected with 100 nM si-NT or 100 nM si-(h)VDAC1, and 24 h post-transfection, cells were fixed and stained with anti-p53 antibodies, followed by secondary Alexa Fluor 488-conjugated anti-rabbit antibodies (shown in green) and DAPI (blue), visualized with a confocal microscope (**D**), arrows point to the nuclear localization), and quantified (**E**). Results show means ± SEM (n = 3), ** *p* < 0.01, *** *p* < 0.001. (**F**) Nuclear and cytosolic fractions were prepared from si-NT- or si-(h)VDAC1-treated cells using a Nuclear/cytosol fractionation kit (Biovision, Milpitas, CA, USA) following the manufacturer’s instructions. Following centrifugation (16,000× *g*, 10 min), the supernatant (cytosolic fraction, Cyto), and pellet (nuclear fraction, Nuc), which was re-suspended in the original volume, were subjected to immunoblotting for KI-67 (nucleus marker), VDAC1 (cytosol fraction) and p53. Total refers to the sample collected prior centrifugation. The original WB image is in the Supplementary Materials.

Cytosolic p53 has been implicated in the induction of mitochondrial outer membrane permeabilization (MOMP) [75], leading to the release of pro-apoptotic factors and inhibition of autophagy [15]. To investigate the effect of VDAC1 expression on the subcellular localization of p53, we expressed p53-GFP in cells and labeled the mitochondria with MitoTracker red. Fluorescence microscopy revealed a substantial fraction of p53 co-localizing with the mitochondria, indicating that a significant fraction of p53 is associated with the mitochondria (Figure 2A).

Perinuclear mitochondrial clustering observed in HeLa cells (Figure 2A) has been previously reported for both HeLa and other cell lines [76]. This clustering tends to increase under stress or during apoptosis. In our case, the use of a transfection reagent for p53 expression may have contributed to the enhanced perinuclear localization observed.

To explore the involvement of VDAC1 in the association of p53 with the mitochondria, we silenced VDAC expression using si-RNA specific to VDAC1 in cells expressing endogenous native levels of p53 (Figure 2B,C). Subcellular distribution of p53 was then assessed using immunofluorescence with p53-specific antibodies (Figure 2D,E). In control cells, p53 was distributed across the cytosol, mitochondria, and to a lesser extent, the nucleus (Figure 2D,E). In contrast, in cells with VDAC1 expression reduced by approximately 50%, p53 localization was significantly altered, resulting in a marked increase in nuclear accumulation (Figure 2E).

To further validate the translocation of p53 to the nucleus following VDAC1 silencing, we performed nuclear and cytosolic fractionation of cells with normal and silenced VDAC1 expression (Figure 2F). The results revealed that in cells exhibiting markedly reduced VDAC1 levels, a marked reduction in p53 in the cytosolic fraction (as indicated by VDAC1 expression) and a corresponding increase in p53 accumulation in the nuclear fraction, confirmed by the nuclear proliferation marker KI-67, were obtained (Figure 2F).

These findings suggest that VDAC1 contributes to p53’s mitochondrial localization and may function as a docking platform for it at the OMM. 

### 3.3. p53-Induced Apoptosis Is VDAC1 Dependent

**Figure 3:**
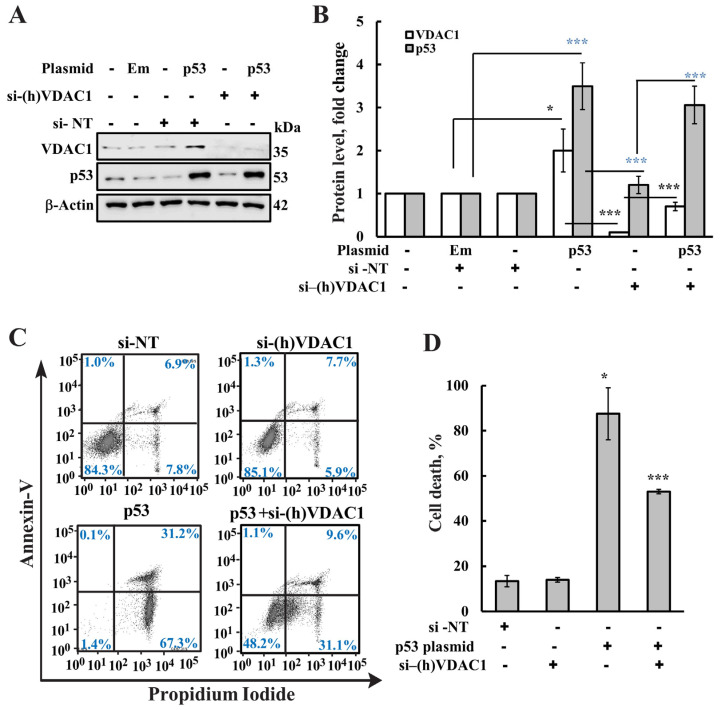
Reducing VDAC1 levels inhibits p53-induced apoptosis. (**A**,**B**) HeLa cells were transfected with si-NT or si-(h)VDAC1 (50 nM), as described in the Methods section and 24 h post transfection, cells were transfected with empty plasmid or pCMV–p53. Approximately 48 h post transfection, VDAC1 and p53 expression levels in control and si-(h)VDAC1-treated cells were analyzed by immunoblotting (**A**) and quantified (**B**). (**C**,**D**) Cell death in the various samples was analyzed using annexin-V-FITC and propidium iodide (PI) staining, and FACS analysis. Representative histograms (**C**) and quantitative analysis of apoptotic cell death (**D**) are shown. Blue and black Asterix refer to significance of control and VDAC1 or control and p53, respectively. Results show means ± SEM (n = 3), * *p* < 0.05, *** *p* < 0.001. The original WB image is in the Supplementary Materials.

To assess whether VDAC1 is required for p53-induced apoptosis, we overexpressed p53 in cells with normal or reduced VDAC1 levels. VDAC1 expression was silenced using specific si-RNA, and the expression levels of VDAC1 and p53 were analyzed by immunoblotting and specific antibodies (Figure 3A,B), and p53-induced apoptosis was assayed by annexin V–FITC and PI staining followed by flow cytometer analysis (Figure 3C,D). In cells expressing endogenous levels of VDAC1, p53 overexpression led to robust apoptosis, with over 85% of cells undergoing cell death. However, in cells with VDAC1 expression reduced by approximately 50%, p53-induced apoptosis was significantly attenuated, with only ~50% cell death observed (Figure 3C,D).

**Figure 4:**
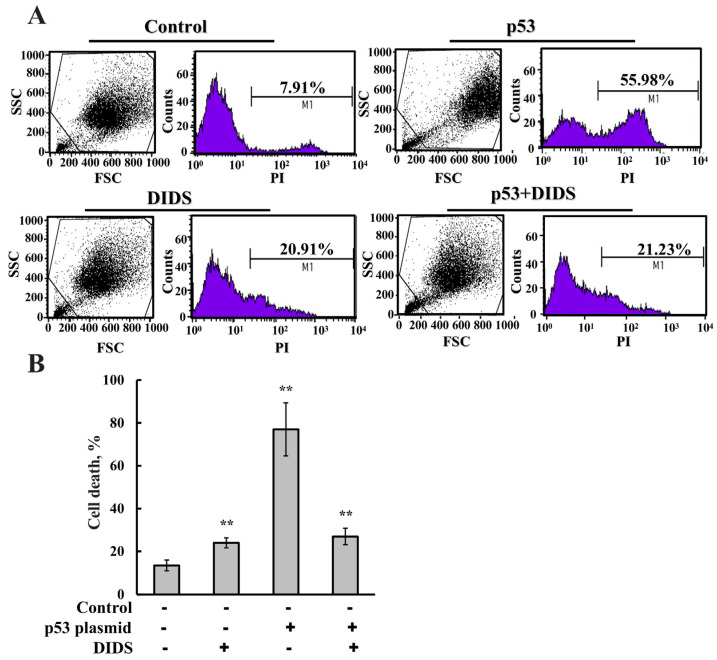
VDAC1 inhibitor DIDS inhibits p53-induced apoptosis. HeLa cells were transfected with empty plasmid or pCMV–p53 and 24 h post transfection, cells were incubated with 70 μM DIDS. After 48 h, cell death was analyzed using PI staining, and FACS analysis. Representative histograms are presented, where the Y−axis (SSC) represents side scatter indicating cell granularity and the X-axis (FSC) represents forward scatter reflecting cell size (**A**). Quantitative analysis of apoptotic cell death (**B**). Results show means ± SEM (n = 3), ** *p* < 0.01.

To further confirm the involvement of VDAC1, we examined the effect of DIDS, a small molecule known to bind VDAC1, inhibit its channel conductance and oligomerization, and suppress apoptosis [39,77,78,79]. Treatment with DIDS markedly reduced p53-induced apoptosis, as determined by annexin V–FITC/PI staining and flow cytometry, bringing cell death levels down to those seen in DIDS-treated control cells (Figure 4A,B).

Together, these results indicate that p53-induced apoptosis is mediated, at least in part, through VDAC1. This finding is in agreement with the findings that mitochondrial p53 exerts pro-apoptotic activity independently of PUMA and Bax [27].

### 3.4. Effect of Purified p53 on the Oligomeric State of Purified and Mitochondria-Embedded VDAC1

**Figure 5:**
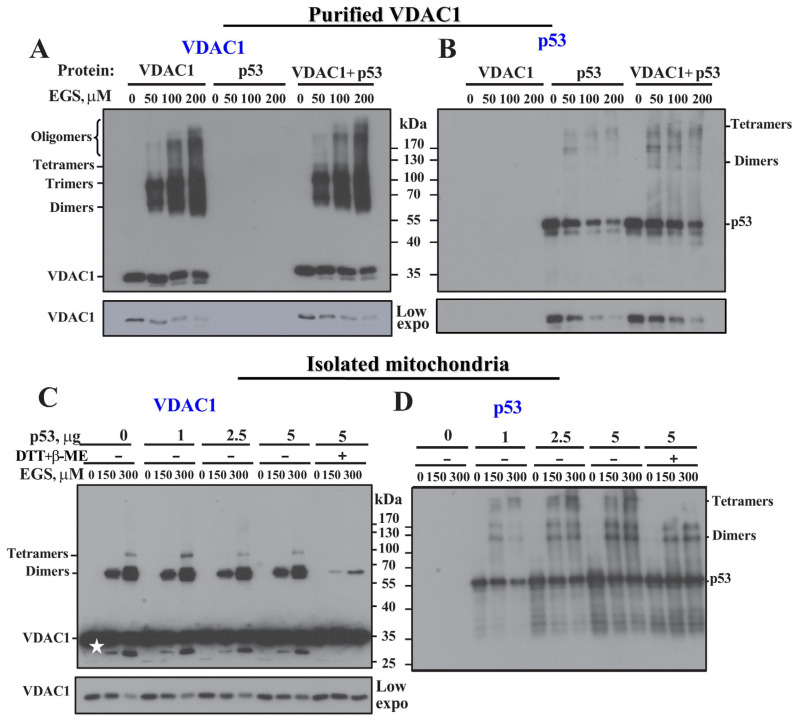
Effect of p53 on purified and mitochondria-embedded VDAC1 oligomeric state. (**A**,**B**) Purified VDAC1 (0.2 μM) was incubated for 5 min with reducing agent free-p53 (2 μM), obtained using the Sephadex-G-50 centrifugation-chromatography method. Then samples were incubated with the indicated concentrations of EGS for 15 min at 30 °C, followed by 10% SDS-PAGE and immunoblotting using anti-VDAC1 (**A**) or anti-p53 (**B**) antibodies. A low exposure is presented at the bottom to demonstrate the decrease in monomeric VDAC1 or p53 upon its crosslinking. (**C**,**D**) Purified rat mitochondria (0.5 mg/mL) were incubated for 15 min at 25 °C with the indicated concentrations of reducing agent free-p53 or without their removal (last 3 lanes), followed by EGS crosslinking and immunoblotting using anti-VDAC1 (**C**) or anti-p53 (**D**) antibodies. The final concentrations of β-mercaptoethanol (β-ME) and dithiothreitol (DTT) were 15 mM and 5 mM, respectively. The VDAC1 band labeled with the asterisk **★** points to intermolecular crosslinked VDAC1 as was previously identified as. The dimer, trimer, tetramer, and oligomer positions, as well as molecular weight standards are also indicated.

To assess whether p53 influences the oligomeric state of VDAC1, we performed chemical crosslinking experiments using purified proteins. As previously reported [42], purified VDAC1 can form stable oligomers that are preserved following crosslinking with EGS. SDS-PAGE analysis of EGS-treated VDAC1 revealed distinct oligomeric species corresponding to dimers (~69 kDa), trimers (~95 kDa), and higher-order oligomers (Figure 5A).

When VDAC1 was incubated with purified p53 prior to crosslinking, no significant change was observed in its oligomeric profile, suggesting that p53 does not affect the formation or stability of VDAC1 oligomers under these in vitro conditions (Figure 5A). Immunoblotting the same membrane with anti-p53 antibodies confirmed the presence of crosslinked p53 dimers, trimers, and tetramers, consistent with p53’s known oligomeric structure (Figure 5B) [80]. No overlapping bands were detected between the anti-VDAC1 and anti-p53 blots, indicating that no stable VDAC1–p53 complexes were formed or preserved by EGS crosslinking under these experimental conditions. This suggests that while p53 and VDAC1 interact (Figure 1), their association may be transient or not sufficiently stable to withstand chemical crosslinking.

It was previously reported [27,29] that incubating isolated mitochondria with purified p53, followed by chemical crosslinking, led to almost the complete disappearance of detectable VDAC1 oligomers. This was interpreted as evidence that p53 promotes the formation of high-molecular-weight VDAC1 complexes that fail to enter the gel during SDS-PAGE analysis [27,29]. To evaluate this observation, we performed similar experiments by incubating isolated mitochondria with increasing concentrations of purified p53 (up to 5 µM), followed by crosslinking with EGS. In contrast to the previous reports, we observed no significant change in the oligomeric pattern of VDAC1. Dimers and trimers were readily detected and stabilized by EGS, regardless of p53 treatment (Figure 5C).

The purified p53 used in our experiments was processed to remove the reducing agents dithiothreitol (DTT) and β-mercaptoethanol, which are typically present in the p53 purification buffer, but can interfere with chemical crosslinking by reacting with the crosslinker EGS. It is well established that reducing conditions are essential for p53 stability since, under oxidative conditions, p53 tends to shift from its functional tetrameric form to monomers and high-molecular-weight aggregates (>1000 kDa) [81].

As shown in Figure 5C (last three lanes), when reducing agents were not removed from the p53 solution, a marked decrease in the levels of crosslinked VDAC1 dimers and trimers was observed. Similarly, the formation of high-molecular-weight p53 oligomers was inhibited under reducing conditions (Figure 5D). These results demonstrate that effective crosslinking of VDAC1 occurs only when p53 is free of reducing agents, and that the presence of DTT or β-mercaptoethanol interferes with EGS-mediated crosslinking of both p53 and VDAC1 (Figure 5C).

Our explanation for the reported effect of p53 on VDAC1 oligomerization is that the reported disappearance of VDAC1 oligomers [27,29] is not due to the formation of high-molecular gel non-penetrating complexes, but rather to the limited amount of the crosslinker because reducing agents in the reaction likely interacted with it and quenched the crosslinking reaction (Figure 5C). This results in a lack of detectable VDAC1 and p53 crosslinked products; thus, leading to a lack of detectable oligomeric products. Moreover, careful evaluation of the data presented in Figure 1A of reference [29] clearly show that, in the presence of p53, the level of monomeric VDAC1 appears to increase, rather than decrease, in the presence of p53, while no high-molecular-weight species are observed at the top of the gel (see Figure 1C in [29]). 

In light of these observations, and contrary to previous claims, we conclude that under properly controlled, reducing agent-free conditions, purified p53 does not significantly alter the oligomeric state of mitochondria-associated VDAC1

### 3.5. Overexpressing p53 Enhances VDAC1 Expression and Oligomerization 

**Figure 6:**
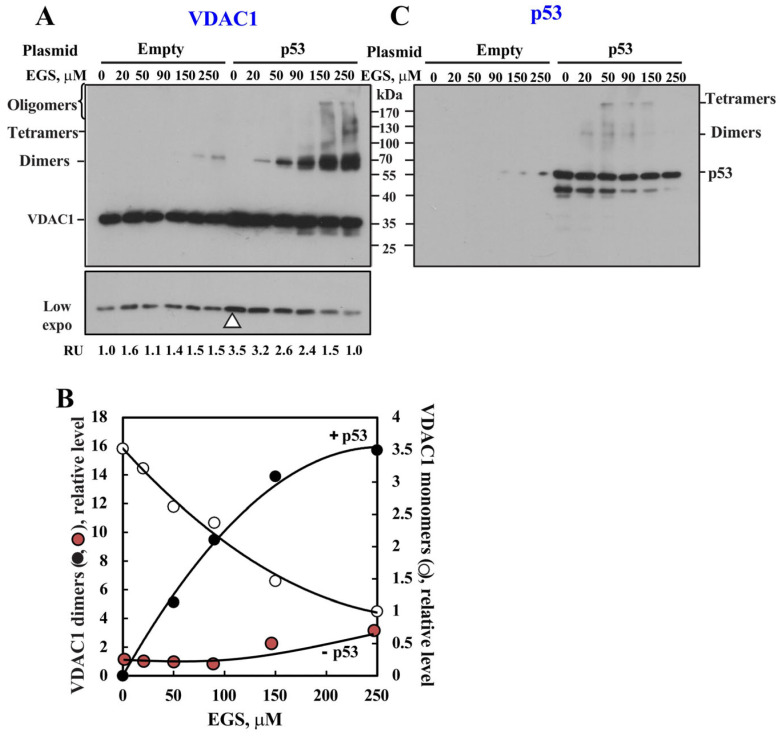
Overexpression of p53 in HeLa cells enhances VDAC1 expression and oligomerization. HeLa cells were transfected with empty or pCMV–p53 (2 μg) plasmid. Approximately 24 h post transfection, cells were analyzed for VDAC1 oligomerization (**A**,**B**) and p53 expression (**C**). Cells were harvested, washed with PBS, and incubated (3 mg/mL) with the indicated concentrations of EGS at 30 °C for 15 min and then subjected to SDS−PAGE and immunoblotting using anti−VDAC1 (**A**) or anti−p53 (**C**) antibodies. A low exposure is presented at the bottom, to demonstrate the increase in VDAC1 levels in p53-expressing cells (shown by the white arrowhead) and the decrease in monomeric VDAC1 upon its crosslinking (**A**). The positions of VDAC1 monomers to multimers and of molecular weight standards are indicated. Quantitative analysis of VDAC1 monomer and dimer levels is presented in relative units (RUs) as a function of EGS concentration (**B**).

We next investigated the effect of p53 expression on VDAC1 oligomerization in HeLa cells (Figure 6), which lack functional p53 due to its degradation by the human papilloma virus E6 protein [82]. Cells were transfected with a p53-expressing construct, then subjected to chemical crosslinking using increasing concentrations of EGS. VDAC1 monomer and oligomer levels were assessed by immunoblotting with anti-VDAC1 antibodies (Figure 6). Overexpression of p53 markedly enhanced VDAC1 oligomerization, leading to the appearance of dimers and higher-order complexes (Figure 6A). A quantitative analysis revealed up to a 16-fold increase in VDAC1 dimers in p53-expressing cells, and only a 2-fold increase in dimers in cells transfected with an empty vector (Figure 6B). As expected, concomitant with the increase in VDAC1 oligomer formation, monomeric VDAC1 levels had decreased (Figure 6A,B). p53 expression and its EGS-mediated crosslinking also demonstrated its oligomerization (Figure 6C), with no common immunoreactive band detected with both anti-VDAC1 and anti-p53.

Additionally, p53 expression also led to an approximately 3.5-fold increase in monomeric VDAC1 expression, as indicated by the arrowhead in Figure 6A and quantified in relative units (RUs) at the bottom of the blot.

### 3.6. p53 Induces VDAC1 Overexpression and Oligomerization

**Figure 7:**
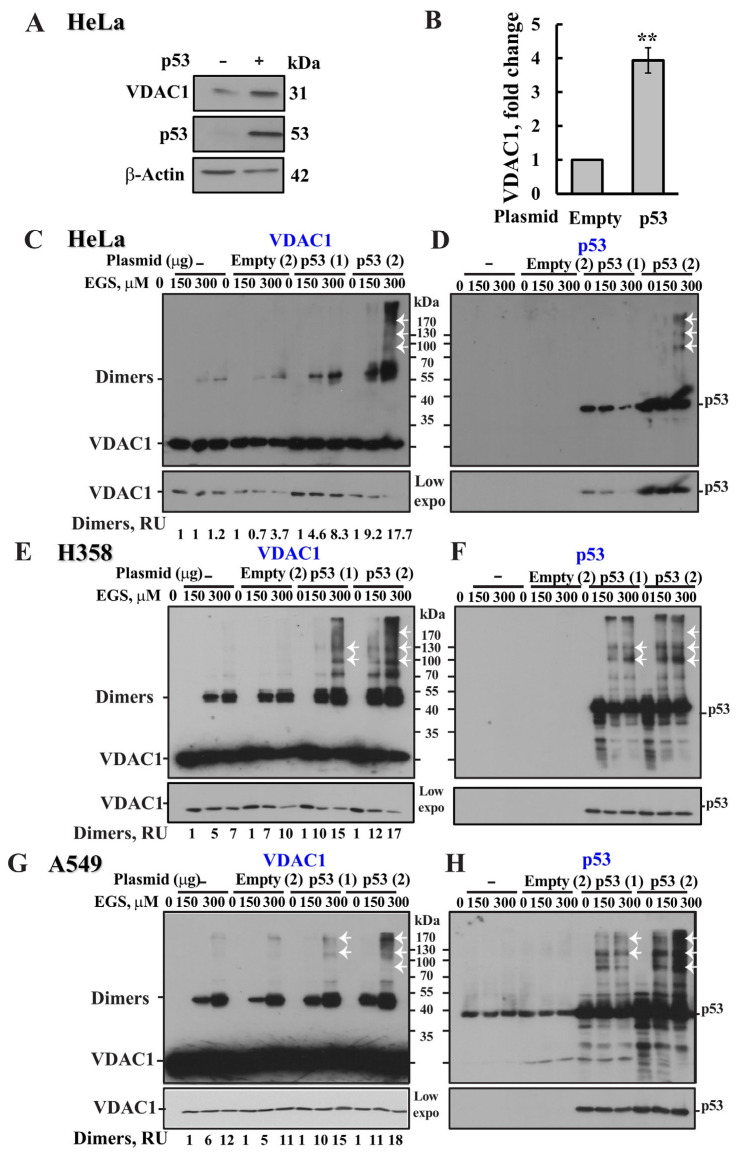
p53 expression induces VDAC1 overexpression and oligomerization in several cell lines. HeLa cells were transfected with either empty pCMV vector or a pCMV–p53 expression plasmid (2 μg) to overexpress p53. Cells were analyzed 24 h post transfection. VDAC1 and p53 expression levels were analyzed by immunoblotting and anti−VDAC1 and anti−p53 antibodies (**A**). Quantitative analysis of VDAC1 expression levels (**B**) correspond to the mean ± SE (n = 2–6). HeLa (**C**,**D**), H358 (**E**,**F**), and A549 (**G**,**H**) cells were transfected with empty (2 μg) or pCMV–p53 plasmid (1 or 2 μg). 24 h post transfection, cells were analyzed for VDAC1 (**C**,**E**,**G**) and p53 (**D**,**F**,**H**) oligomerization using the indicated concentrations of EGS and immunoblotting using anti−VDAC1 or anti−p53 antibodies. A low exposure is presented at the bottom of each blot to demonstrate the decrease in monomeric VDAC1 upon its crosslinking. Quantitative analysis of VDAC1 dimers levels is presented in relative units (RUs) (**C**,**E**,**G**). The white arrows point to VDAC1- and p53-containing complexes. The positions of VDAC1 monomers to multimers and of molecular weight standards are indicated. Results show means ± SEM (n = 3), ** *p* < 0.01.

As p53 expression led to increased levels of monomeric VDAC1 (Figure 6A, arrowhead), we hypothesized that p53 enhances VDAC1 oligomerization by upregulating VDAC1 expression. Accordingly, the effect of p53 expression on VDAC1 levels was examined in HeLa cells (Figure 7A,B). Immunoblot analysis (Figure 7A) revealed that p53 overexpression significantly increased VDAC1 protein levels (approximately 4-fold, Figure 7B), suggesting that p53 may regulate VDAC1 expression by acting as a transcription factor.

We assessed the impact of p53 expression on VDAC1 oligomerization not only in HeLa cells, but also in p53 null lung carcinoma, H358 cells and p53 expressing lung carcinoma, A549 cells (Figure 7C–H). In HeLa, H358 and A549 cell overexpression of p53 led to a pronounced increase in oligomer formation, as shown by immunoblotting following chemical crosslinking (Figure 7C,E,G). A quantitative analysis showed high increases in VDAC1 dimer formation when p53 was highly increased (Figure 7D,F,H). Concomitant with the increase in the levels of VDAC1 oligomers, the levels of monomeric VDAC1 decreased (Figure 7C,E,G, see low exposure), as expected from its crosslinking.

Immunoblotting the same samples with anti-p53 antibodies detected multiple high-molecular-weight p53-containing complexes (Figure 7D,F,H), consistent with p53’s known tetrameric structure [83]. Interestingly, several of these high mass complexes appeared to be immunoreactive with anti-VDAC1 antibodies (indicated by arrows in Figure 7C–H), suggesting the possible formation of crosslinked VDAC1–p53 complexes.

## 4. Discussion

The tumor suppressor p53 functions not only as a “guardian of the genome,” but it also plays a crucial role in regulating mitochondrial integrity and functions, including apoptosis [84]. Under stress conditions, p53 levels increase and its tetrameric form specifically binds to the promoters of target genes [85]. This tetrameric state of p53 is essential for DNA binding [73]. However, p53 can assemble into various oligomeric forms, ranging from functional tetramers and octamers that bind DNA to inactive amyloid-like structures, including fibrils and amorphous aggregates. These distinct oligomeric states influence cell fate decisions—such as proliferation, growth arrest, or apoptosis—and are central to p53’s role in cancer biology [86,87].

p53 function have primarily been attributed to its ability to trigger apoptosis in response to genotoxic stress by acting as a transcription factor that upregulates pro-apoptotic members of the Bcl-2 family, such as Bax, Bid, Noxa, and PUMA [88,89,90,91]. However, p53 can also induce apoptosis independently of protein synthesis [25,92]. Transcriptionally inactive p53 mutants are still capable of promoting apoptosis in certain cell types [93], indicating a transcription-independent pro-apoptotic role for p53. Furthermore, a fraction of p53 can localize to the mitochondria in tumor cells undergoing DNA damage-induced apoptosis [19], where it directly activates mitochondrial apoptotic pathways.

In the mitochondria, p53 interacts with several mitochondrial proteins [27,28,88,89,90,91] including Bak promoting its oligomerization and Cyto *c* release [28]. p53 also induces Bax activation [94], disrupting Bcl-2–Bax interactions by binding Bcl-2, thereby enhancing Bax-mediated apoptosis [95]. Importantly, studies using mitochondria isolated from *PUMA*^–/–^ or *Bax*^–/–^ mice have shown that p53 translocated to the mitochondria induces the release of Cyto *c*, SMAC, and AIF and cell death independent of PUMA and Bax [27].

**Figure 8:**
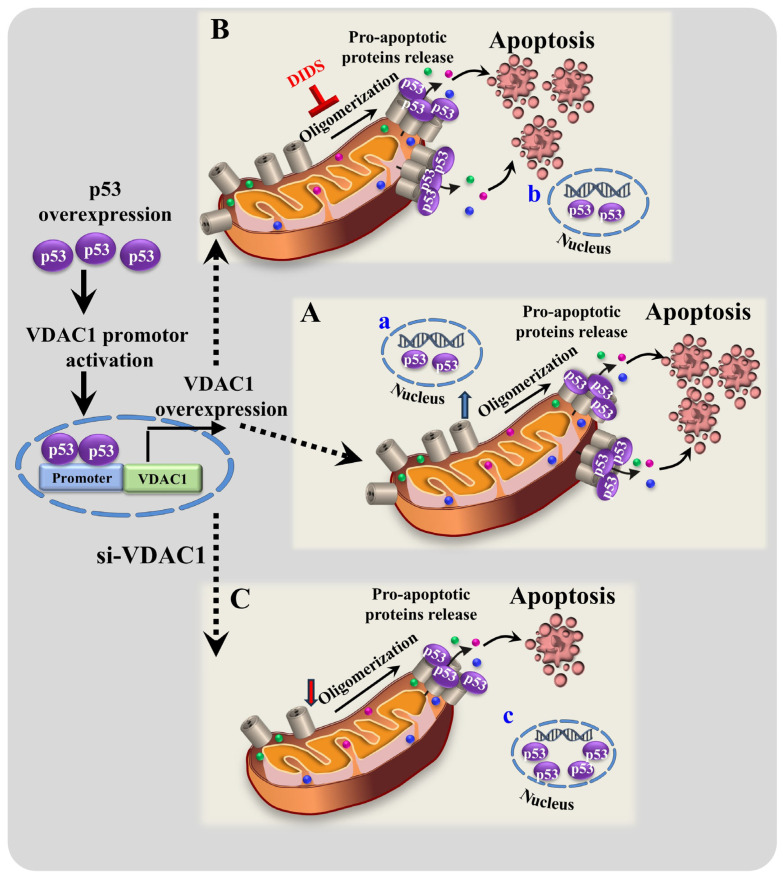
Proposed model for p53 increases VDAC1 expression level and induces apoptosis. (**A**) Overexpression of p53 leads to activation of the VDAC1 promoter, resulting in increased VDAC1 gene expression. p53 is present and mainly mitochondrial bound, with a fraction found in the nucleus (**a**). (**B**) p53-induced mitochondria-mediated apoptosis via enhancing VDAC1 expression level and, subsequently, VDAC1 oligomerization, allowing pro-apoptotic protein release from the inter-mitochondrial space, leading to apoptotic cell death. DIDS inhibits VDAC1 oligomerization and, subsequently, p53-induced apoptosis. p53 is present and mainly mitochondrial bound, with a fraction found in the nucleus (**b**). (**C**) VDAC1 expression level influences the subcellular localization of p53. si-(h)VDAC1 decreased VDAC1 levels, reduced p53-induced VDAC1 oligomers and apoptosis, and led to nuclear accumulation of p53 (**c**), suggesting that VDAC1 regulates its mitochondrial trafficking.

In the present study, summarized in Figure 8, we demonstrated that p53 expression led to increased levels of VDAC1, its oligomerization, and the induction of apoptosis (Figure 3, Figure 4, Figure 5, Figure 6 and Figure 7). p53-mediate VDAC1 oligomerization and apoptosis were highly reduced when VDAC1 expression was silenced using specific si-RNA (Figure 3). Moreover, DIDS—a known inhibitor of VDAC1 channel conductance, oligomerization, Cyto *c* release [39,79,96], and apoptosis induced by various stimuli [77,78,96]—significantly inhibited p53-induced apoptosis (Figure 4).

While nuclear p53 functions primarily as a DNA-binding tetramer that regulates the transcription of genes involved in cell-cycle arrest and apoptosis, p53 also exhibits important transcription-independent roles in the cytoplasm. In the cytosol, it exists in monomeric, dimeric, or oligomeric forms, and participates in non-transcriptional processes, including the regulation of metabolism, inhibition of autophagy, and promotion of both apoptosis and necrosis [2,3,4,15,29,97].

These cytoplasmic p53 oligomers may differ structurally from nuclear forms due to post-translational modifications (e.g., phosphorylation, ubiquitination) and interactions with cytosolic proteins, such as members of the Bcl-2 family [19]. Here, we found that p53 directly interacts with VDAC1 and modulates its channel conductance (Figure 1). Our findings show that silencing VDAC1 did not significantly affect the levels of p53 but altered its subcellular distribution with p53 translocation and accumulation in the nucleus (Figure 2).

Similar results were observed in glioblastoma multiforme (GBM) tumors treated with siRNA targeting VDAC1, where p53 was upregulated and accumulated in the nucleus [98].

This suggests that VDAC1 serves as a mitochondrial docking site for p53 and may play a key role in modulating p53 localization and function.

These results imply that VDAC1 is essential in p53-induced apoptosis, with p53 acting as a transcription factor that upregulates VDAC1 expression. The increased VDAC1 levels, in turn, promote its oligomerization—a step that is critical in the apoptotic process [37,38,39,40,41,42]. The enhanced VDAC1 oligomerization by p53 may also be supported by a direct interaction between p53 and VDAC1, showing cooperative interaction between the two proteins (Hill coefficient = 3–4), suggesting their interaction at an oligomeric state (Figure 1).

However, although some VDAC1/p53-containing complexes were obtained in cells transformed to overexpress p53 (Figure 5, Figure 6 and Figure 7), no such complexes were obtained with either purified VDAC1 or mitochondrial membrane-embedded VDAC1 (Figure 5). These findings contrast with earlier studies that used isolated mitochondria, showing that VDAC1-containing complexes (60–300 kDa) seen in control mitochondria disappeared upon p53 treatment [27,29], and may indicate that, in the presence of p53, the high-molecular-weight complexes of VDAC1 failed to enter the gel. This discrepancy between our results and these reported findings may be explained by the presence of reducing agents (e.g., β-mercaptoethanol or DTT), which can interfere with crosslinking reactions. Supporting this idea, our results show that when p53 was not free of reducing agents, its ability to promote VDAC1 oligomerization was markedly reduced (Figure 5C,D). Moreover, previously published studies [see Figure 1C in [29]] reported that p53 actually increased monomeric VDAC1 levels, rather than decreasing them, apparently upon its crosslinking, with no accumulation of high-molecular-weight VDAC1 oligomers at the top of the gel.

p53 is susceptible to oxidation, which prevents it from binding to its DNA response element. Reducing agents such as DTT enhance its binding to DNA, likely by preserving reactive cysteine thiols that are critical for binding to DNA [99,100]. Moreover, oxidation was found to increase the level of monomeric and very high-molecular-weight p53 oligomers (>1000 kDa), at the expense of the functional tetrameric form [81].

Taken together, these findings suggest that while p53 enhances VDAC1 expression and oligomerization leading to apoptosis, this process likely involves mechanisms beyond direct interaction with VDAC1 to drive its oligomerization.

Based on our results showing that p53 induces VDAC1 overexpression and subsequent oligomerization and cell death (Figure 6 and Figure 7), we propose a novel mechanism (Figure 8) in which p53 first enhances VDAC1 expression, leading to its oligomerization. These VDAC1 oligomers form channels for pro-apoptotic protein release [37,38,39,40,41,42], ultimately triggering apoptotic cell death (Figure 3C,D and Figure 4). The observed increase in VDAC1 levels is consistent with p53’s role as a transcription factor [88,89,90,91] and in accordance with p53 being implicated in the regulation of VDAC1 expression in neuronal cells via pathways associated with ATP depletion and metabolic oxidative stress [65].

In this context, VDAC1 overexpression has been shown to be a critical determinant of mitochondria-mediated apoptosis. Multiple studies have reported that various apoptosis-inducing stimuli, acting through different signaling pathways, also lead to increased VDAC1 expression [32,41,44,45,46,101,102,103].

### Interaction Between p53 and VDAC1: Implications for Mitochondrial Function and Cell Fate

The interaction between p53 and VDAC1, as well as their mutual regulation, plays a pivotal role in determining cell fate under stress conditions. A growing body of evidence supports a direct role for p53 in modulating VDAC1-mediated apoptotic pathways. For instance, p53 has been shown to contribute to mitochondrial membrane permeabilization, promoting apoptosis through VDAC1-dependent mechanisms [104].

Mechanistically, p53 can disrupt the interaction between hexokinase 2 (HK2) and VDAC1 by promoting VDAC1 phosphorylation through the activation of glycogen synthase kinase 3β (GSK3β). This post-translational modification weakens HK2’s mitochondrial binding, thereby facilitating apoptosis [105].

Beyond post-translational regulation, p53 also acts at the transcriptional level. Under metabolic stress, p53 has been shown to upregulate *VDAC1* gene expression via pathways involving AMPK and PGC-1α, particularly in response to decreased ATP levels and oxidative stress in neuronal cells [65].

Interestingly, the relationship between p53 and VDAC1 is bidirectional. Loss of VDAC1 expression has been found to increase p53 levels without apoptosis activation. Instead, this upregulation of p53 was associated with cancer cell differentiation, suggesting that VDAC1 may act as a switch between apoptotic and non-apoptotic roles of p53 depending on the cellular context [106].

Moreover, peptides derived from VDAC1-interacting proteins such as actin, gelsolin, or GAPDH significantly elevate p53 expression levels and promote apoptosis [36].

Collectively, these findings underscore a complex and dynamic interplay between p53 and VDAC1, integrating metabolic cues, stress responses, and cell fate decisions—highlighting VDAC1 as not only a downstream effector of p53 but also a modulator of its function.

## 5. Conclusions

In conclusion, our findings, summarized in Figure 8, suggest that p53 induces mitochondria-mediated apoptosis by increasing VDAC1 expression, which then undergoes oligomerization to form a pro-apoptotic release channel. This apoptotic pathway can be effectively inhibited by VDAC1-targeting agents such as DIDS, which block both VDAC1 oligomerization and apoptosis or by silencing VDAC1 expression. This mechanism is further supported by our recent study showing that peptides derived from VDAC1-interacting proteins elevate both p53 and VDAC1 expression, leading to VDAC1 oligomerization and cell death.

## Figures and Tables

**Table 1:** Antibodies used in this study. Antibodies against the indicated protein, their catalogue number, source, and the dilutions used in immunoblotting (WB) and immunofluorescence (IF) experiments are listed.

Antibody	Source and Cat. No.	Dilution	
		**WB**	**IF**
Rabbit monoclonal anti-VDAC1	Abcam, Cambridge, UK, ab154856	1:2000	1:500
Mouse monoclonal anti-p53	Santa Cruz Biotechnology, Santa Cruz, CA, USA, sc-126	1:10,000	1:300
Rabbit monoclonal anti-KI-67	Abcam; Cambridge, UK, ab16667	1:1000	-
Mouse monoclonal anti-β-actin	Millipore, Billerica, MA, USA, MAB1501	1:10,000	-
Goat anti-rabbit (Alexa Fluor 488)	Abcam, Cambridge, UK, ab150077	-	1:400
Goat anti-rabbit (HRP)	Promega, Madison, WI, USA, W4018	1:15,000	-
Donkey anti-mouse (HRP)	Abcam, Cambridge, UK, ab98799	1:10,000	-

## Data Availability

The original contributions presented in this study are included in the article/Supplementary Material. Further inquiries can be directed to the corresponding author.

## Supplementary Materials

The following supporting information can be downloaded at: https://www.mdpi.com/article/10.3390/biom16010141/s1, Figure S1: Original blots.

## Abbreviations

The following abbreviations are used in this manuscript:

Cyto *c*	cytochrome *c*
EGS	ethylene glycol bis[succinimidylsuccinate]
OMM	outer mitochondrial membrane
PLB	planar lipid bilayer
VDAC	voltage-dependent anion channel
