# Supplementary figures and images for: p53 Interacts with VDAC1, Modulating Its Expression Level and Oligomeric State to Activate Apoptosis
Source: Biomolecules. 2026 Jan 13;16(1):141. doi: 10.3390/biom16010141 (PMC12838997; doi:10.3390/biom16010141)

## Original Blots

Fig.2F

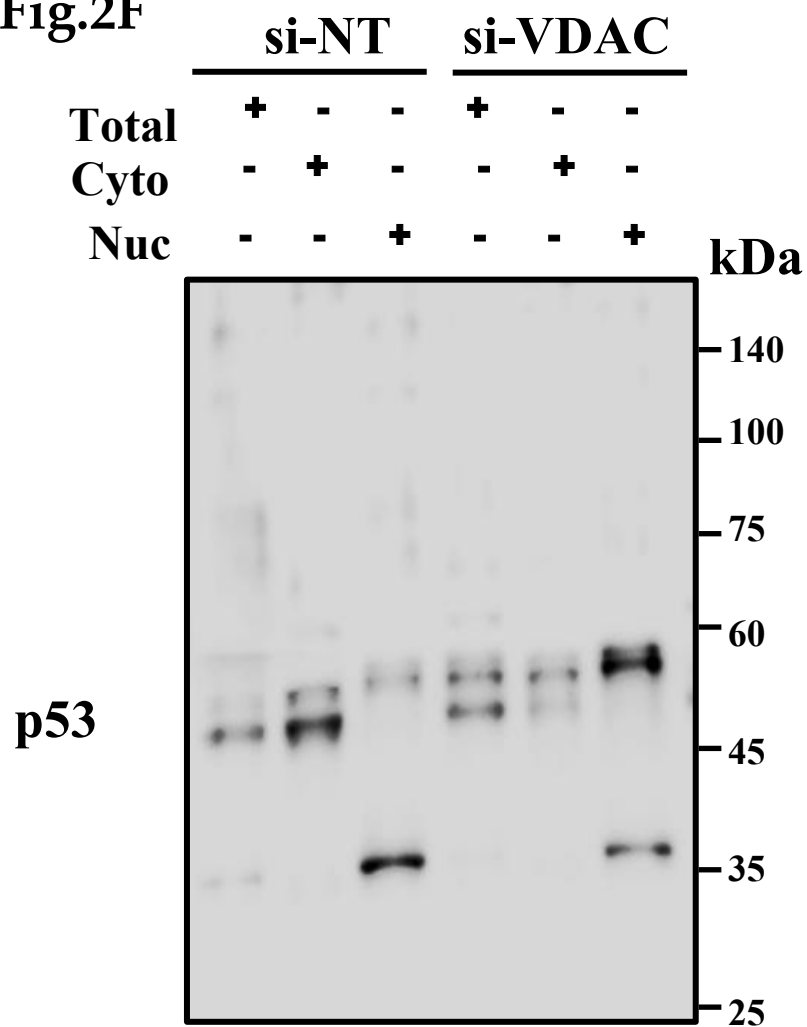

Fig.2F

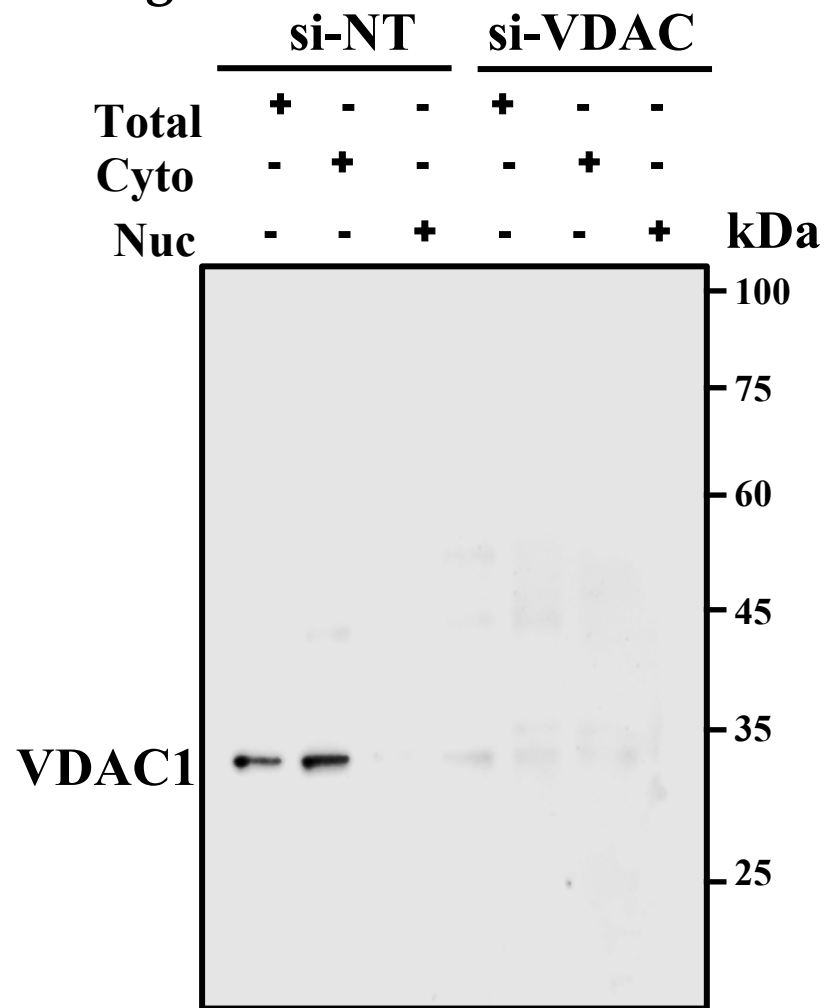

Fig.2F

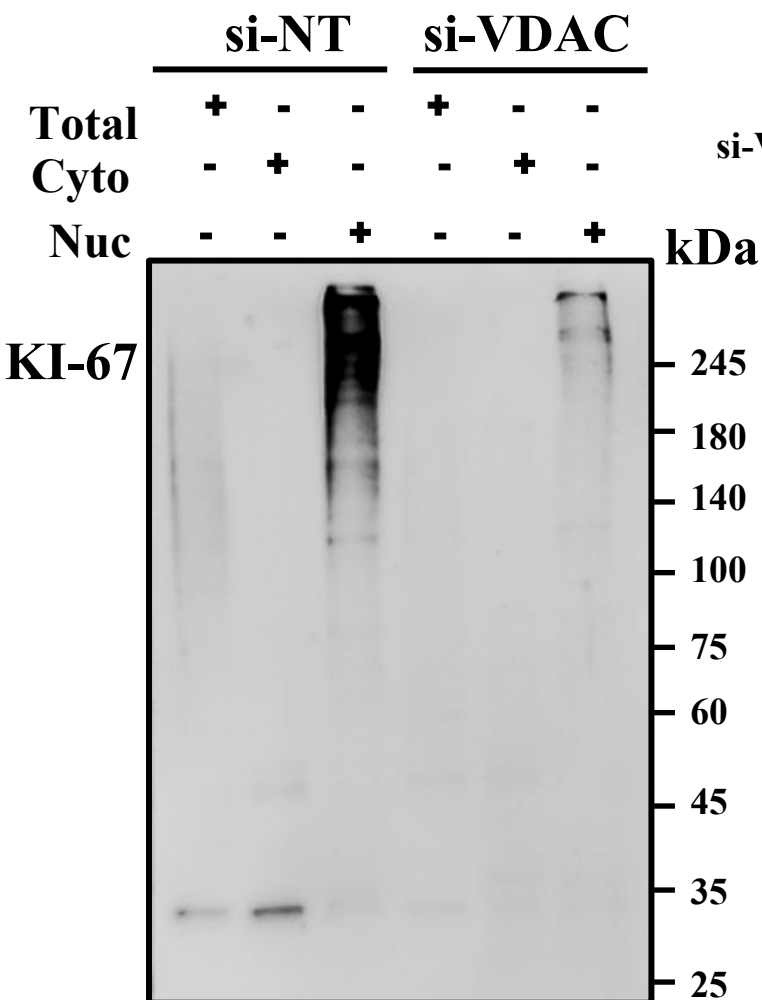

Fig. 2 B

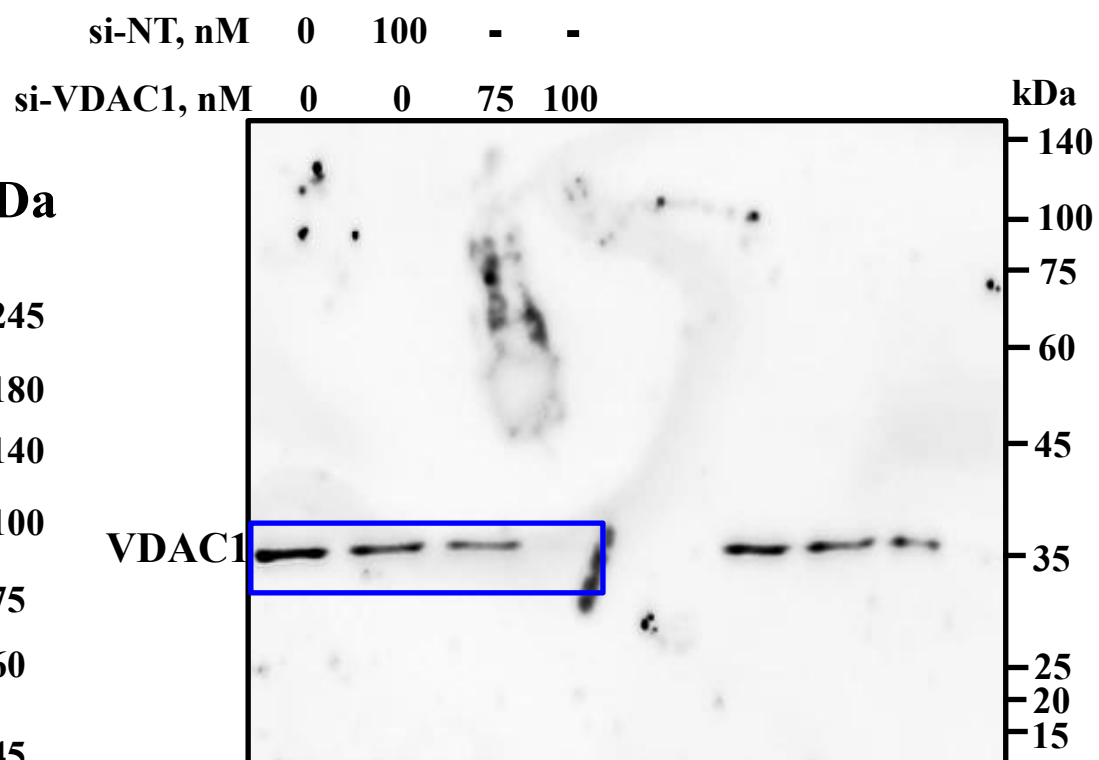

Fig. 3A

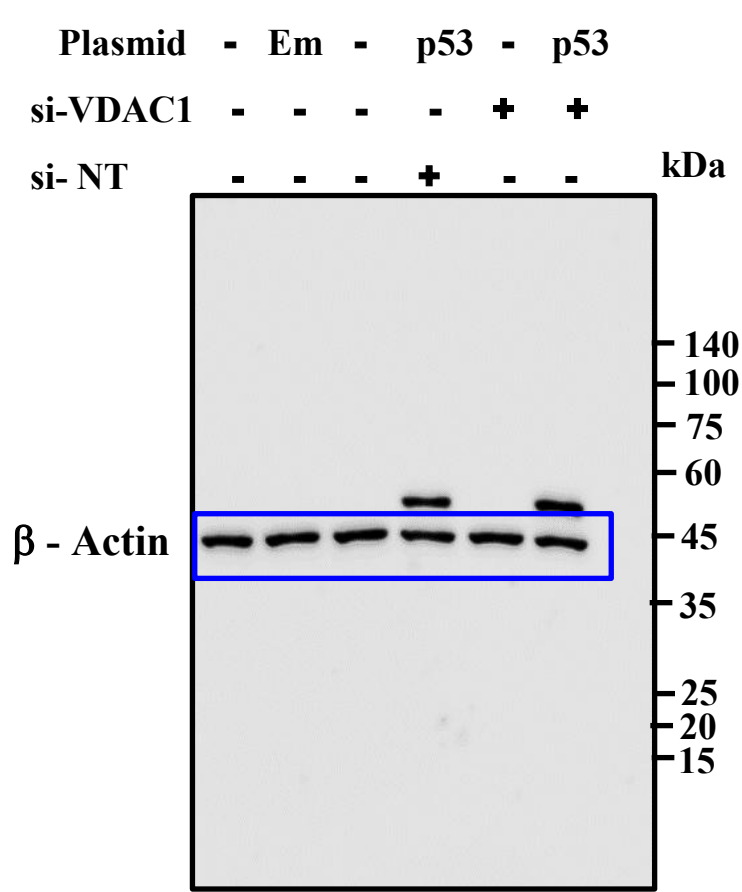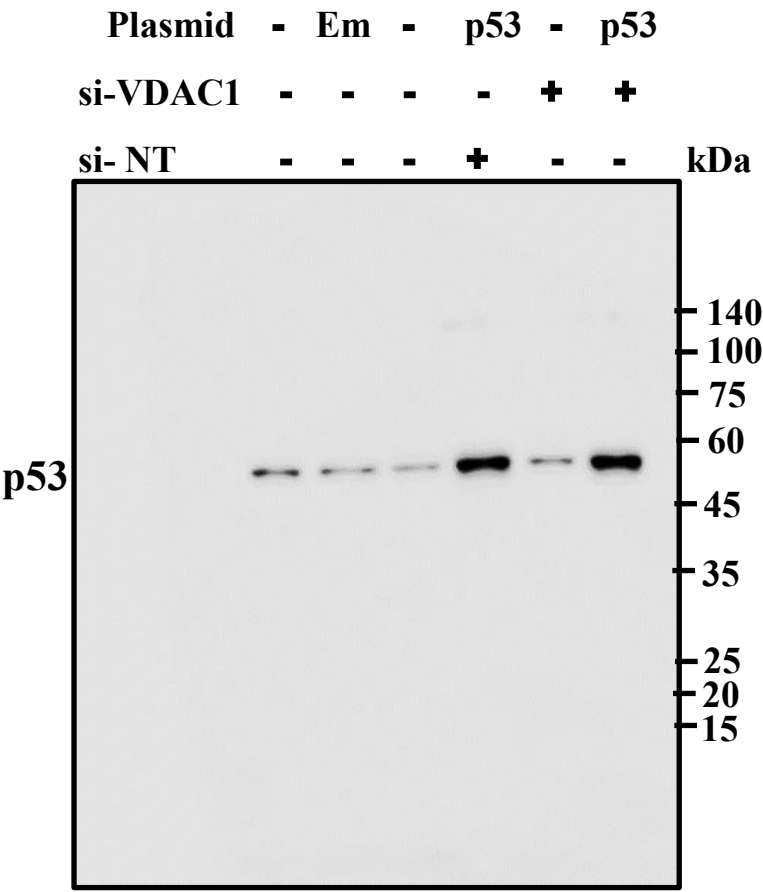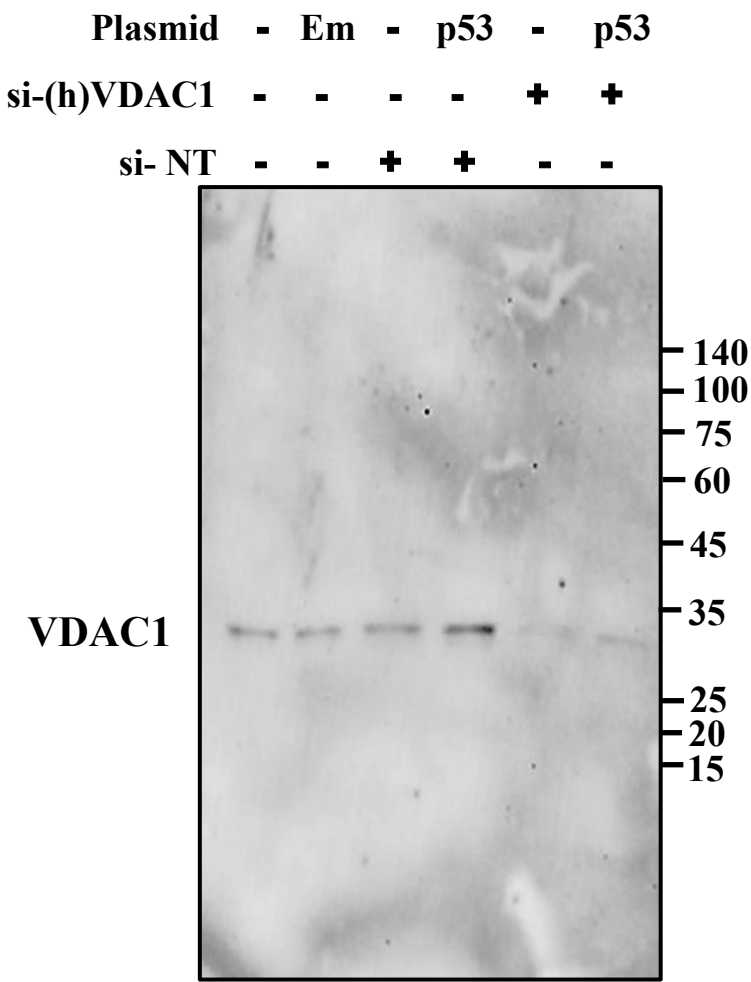

**Fig. 7A**

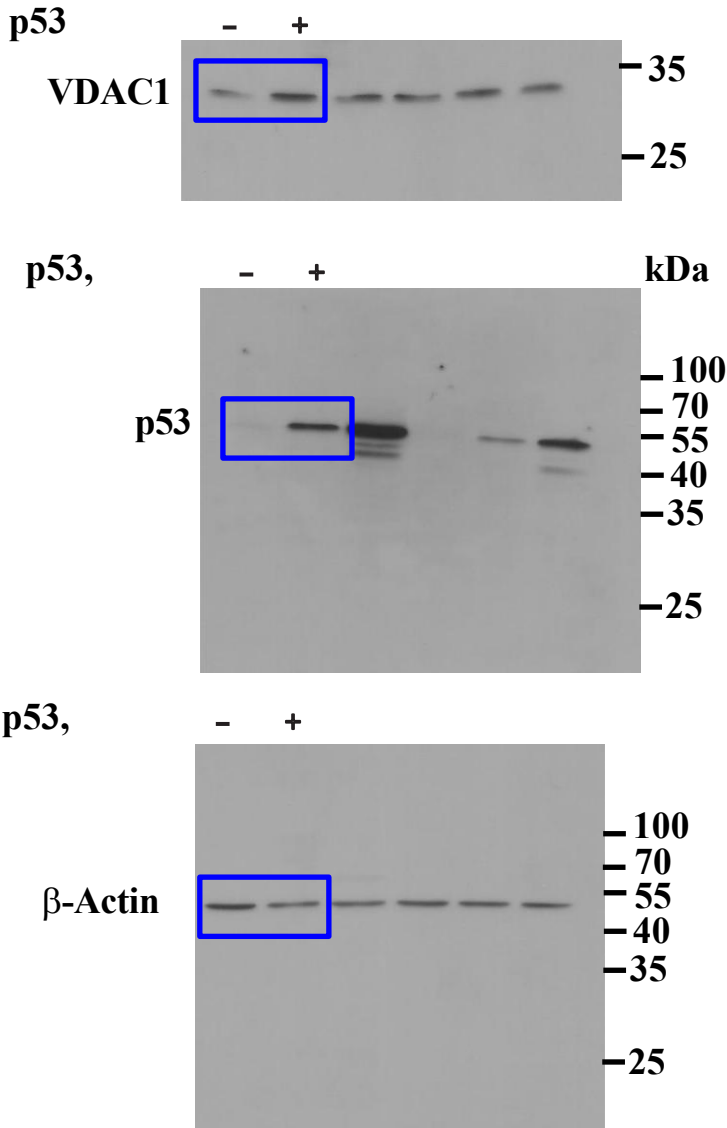

Supplement: Supplementary file 1 [file biomolecules-16-00141-s001.zip › biomolecules-3970279-supplementary.pdf]
